# A good sugar, d-mannose, suppresses autoimmune diabetes

**DOI:** 10.1186/s13578-017-0175-1

**Published:** 2017-09-25

**Authors:** Yun-Bo Shi, Deling Yin

**Affiliations:** 1 Molecular Morphogenesis, National Institute of Child Health and Human Development, National Institutes of Health, Bethesda, MD USA; 2 Department of Internal Medicine, College of Medicine, East Tennessee State University, Johnson City, TN USA

**Keywords:** Glucose, Mannose, Diabetes, Autoimmune disease, Inflammation, Immunosuppression

## Abstract

It is well known that too much sugar uptake causes many health problems, including diabetes and obesity (Lustig et al. in Nature 482:27–29, 2012). However, a team of researchers led by Dr. Wanjun Chen of the National Institute of Dental and Craniofacial Research (NIDCR), National Institutes of Health (NIH), USA, have recently shown that d-mannose, a naturally occurring C-2 epimer of glucose is likely beneficial to human health. Their studies have revealed that supraphysiological levels of d-mannose that are safely achievable via drinking-water supplementation can be preventive and therapeutic to experimental autoimmune diabetes and asthmatic lung inflammation (Zhang et al. in Nat Med 23:1036–1045, 2017).

With the improvement of the living standard in the world, the incidence of many health problems has significantly increased over the past few decades. Among them include autoimmune diseases, obesity, diabetes, cardiovascular and cerebrovascular diseases, allergy/asthma, and cancer. In addition to the genetic and environmental factors, diet has long been postulated as a potential risk factor for the emerging and increase of many of aforementioned diseases in the world, especially in the developed countries. One dietary factor, which has rapidly changed along with the western diet and increased consumption of processed food, is sugar [1]. Eating too much sugar raises the risk for health problems that are associated with obesity, diabetes, cardiovascular diseases, and autoimmune diseases, etc. This has led to a logical and popular advice of restriction of sugar uptake.

This notion is not without exception, at least for d-mannose, a monosaccharide and C-2 epimer of glucose, based on new findings from a team of investigators led by Dr. Wanjun Chen of NIH, USA, including his collaborators from China. They have discovered that d-mannose can surprisingly prevent and suppress type 1 autoimmune diabetes and asthmatic lung inflammation [2]. They showed that oral administration of supraphysiological amounts of d-mannose in drinking water of non-obese diabetic (NOD) mice before they developed hyperglycemia could prevent diabetes development in those mice. Strikingly, they also found that oral administration of d-mannose was able to block the progress of diabetes even in NOD mice with new-onset diabetes. Importantly, they also revealed that this d-mannose-mediated suppression of inflammation is not unique in autoimmune diabetes since oral administration of d-mannose also prevented and suppressed airway inflammation in the lungs in an ovalbumin-induced asthmatic lung airway inflammation model. Mechanistically, Dr. Chen and his colleagues have demonstrated that d-mannose induces the generation of regulatory T cell (Treg) from naïve CD4^+^ CD25^**−**^ T cells. Treg cells are essential immunoregulatory cells that are instrumental in the induction and maintenance of immune tolerance and in the prevention and suppression of inflammation and autoimmune diseases. They have further revealed that the d-mannose-mediated generation of Treg cells is accomplished by the activation of transforming growth factor beta (TGF-β), one of the most important immunosuppressive cytokines [3]. The activation of TGF-β in T cells by d-mannose is attributable to at least two independent yet complementary pathways, integrin αvβ8 and reactive oxygen species (ROS) pathways.

These novel findings led the authors to consider their potential clinical implications and the possibility and barriers to overcome in order to translate them into clinical use toward human diseases. While the physiological level of d-mannose in human and mouse blood is relative low (~ 100 μM, 1/50 of the glucose level), it has been reported that it could be increased up to nine-fold in mice with no adverse consequences after long-term d-mannose oral administration [4]. More importantly, stable serum d-mannose levels of up to 2 mM can be reached and are well tolerated in humans without any sign of liver or renal toxicity [5]. More importantly, Dr. Chen and his colleagues showed that in vitro as low as 1 mM of d-mannose was able to induce Treg cell formation from naive CD4^+^ T cells. Furthermore, d-mannose has already been used for human disease treatment, includes congenital disorders of glycosylation type Ib [6] and bacterial urinary tract infection [7]. All these suggest that the supraphysiological levels of d-mannose in human can be reached by administration of d-mannose for disease prevention and treatment without adverse effect on human.

In sum, the studies by Dr. Chen and his colleagues have discovered that d-mannose is a “healthy/good” monosaccharide and suggest that this unique sugar could be a safe dietary supplement to promote immune tolerance and to treat/prevent human diseases associated with autoimmunity and allergy.
